# Parent–child relationships and offspring’s positive mental wellbeing from adolescence to early older age

**DOI:** 10.1080/17439760.2015.1081971

**Published:** 2015-09-15

**Authors:** Mai Stafford, Diana L. Kuh, Catharine R. Gale, Gita Mishra, Marcus Richards

**Affiliations:** ^a^ MRC Unit for Lifelong Health and Ageing at UCL, 33 Bedford Place, LondonWC1B 5JU, UK; ^b^ MRC Lifecourse Epidemiology Unit, University of Southampton, Southampton, UK; ^c^ Department of Psychology, Centre for Cognitive Ageing and Cognitive Epidemiology, University of Edinburgh, Edinburgh, UK; ^d^ School of Population Health, University of Queensland, Brisbane, Australia

**Keywords:** well-being, longitudinal, birth cohort study, life satisfaction

## Abstract

We examined parent-child relationship quality and positive mental well-being using Medical Research Council National Survey of Health and Development data. Well-being was measured at ages 13–15 (teacher-rated happiness), 36 (life satisfaction), 43 (satisfaction with home and family life) and 60–64 years (Diener Satisfaction With Life scale and Warwick Edinburgh Mental Well-being scale). The Parental Bonding Instrument captured perceived care and control from the father and mother to age 16, recalled by study members at age 43. Greater well-being was seen for offspring with higher combined parental care and lower combined parental psychological control (*p* < 0.05 at all ages). Controlling for maternal care and paternal and maternal behavioural and psychological control, childhood social class, parental separation, mother’s neuroticism and study member’s personality, higher well-being was consistently related to paternal care. This suggests that both mother–child and father–child relationships may have short and long-term consequences for positive mental well-being.

## Introduction

Parent–child relationships are central to psychological development and several studies have shown that suboptimal parenting is an important risk factor for psychological disorder in clinical and representative community samples (Blatt & Homann, 1992; Enns, Cox, & Clara, 2002). Many studies have captured parent–child relationships along two principal dimensions. The parental care dimension reflects a continuum from affectionate, warm, responsive parenting to cold and unresponsive parenting. The demandingness or control dimension reflects the extent to which the parent demands and monitors standards for their child’s conduct (Baumrind, 1991). The concept of parental control has been further distinguished as behavioural and psychological forms of control (Barber, 1996). Behavioural control can provide a structured and predictable environment for the child and encourages their socially acceptable behaviour. But there is a need to balance individual autonomy with conformity to social norms and the association between behavioural control and outcomes may be curvilinear (Barber, 1996). Psychological control refers to parenting that is intrusive and manipulates the child’s emotional development (Barber, 1996). A high level of psychological control has been consistently associated with an elevated risk of psychological disorder among the offspring in adolescence and adulthood (van der Bruggen, Stams, & Bogels, 2008; McLeod, Wood, & Weisz, 2007; Radziszewska, Richardson, Dent, & Flay, 1996; Rodgers, 1996a; Shaw, Krause, Chatters, Connell, & Ingersoll-Dayton, 2004). Behavioural control, on the other hand, has been linked to lower risk of psychological symptoms (Wang, Pomerantz, & Chen, 2007). Despite the large literature relating parental care and control to psychological disorder, studies investigating their links with positive mental well-being are far fewer in number and these are summarised below. Positive mental well-being is now recognised as being multidimensional, having hedonic and eudaimonic aspects, and being more than the absence of psychological disorder. Positive mental well-being and psychological disorder do not necessarily have identical risk factors (Huppert & Whittington, 2003; Ryan & Deci, 2001; Ryff & Singer, 1998). There is therefore a need to know whether parent–child relationships influence positive well-being indicators, such as happiness, life satisfaction and positive psychological functioning, in adolescence and beyond.

Drawing on evidence from literature on the development of psychological disorder as well as positive mental wellbeing, we suggest that socio-relational, psychological and socio-economic pathways may link parental care and control to mental well-being in adulthood. Attachment theory details one such pathway. This suggests that infants who experience caring and responsive relationships with their parents are more securely attached to those parents; are more likely to be securely attached in later relationships; and are at lower risk of psychological disorder (Bowlby, 1988). Rutter (1995) highlighted the particular contribution of bonding with the father figure. High parental care is hypothesised to promote social- and neuro-cognitive development for positive interpersonal relations and behaviour (Richards & Hatch, 2011) as well as positive adult relationships (Rodgers, 1996b). Interpersonal relations, including social support and strong social networks, are important determinants of positive mental well-being (Charles & Carstensen, 2010).

Another pathway is through positive psychological functioning facilitated by the parent. Psychological control can reduce a child’s autonomy and impair the development of self-regulation (Grolnick & Ryan, 1989), whilst behavioural control can increase a child’s competence (Grolnick & Pomerantz, 2009). A combination of intrusive parenting and low parental care is associated with greater psychological inflexibility among the offspring in adolescence (Williams, Ciarrochi, & Heaven, 2012), greater emotion suppression in late childhood (Jaffe, Gullone, & Hughes, 2010) and poorer emotion regulation in early adulthood (Manzeske & Stright, 2009), which can result in fewer positive thoughts and emotions (Gross & John, 2003). The development of personality traits may also be implicated, since parental care and control have been associated prospectively with adult offspring’s optimism, empathy and expressiveness (Huppert, Abbott, Ploubidis, Richards, & Kuh, 2010; Johnson, Liu, & Cohen, 2011; Korkeila et al., 2004), though it is recognised that the relationship between parenting practices and child’s personality is likely to be bidirectional (van Zeijl et al., 2007). Optimism and neuroticism are highly correlated with positive mental well-being (Gale, Booth, Mõttus, Kuh, & Deary, 2013; Huppert et al., 2010).

Finally, socio-economic pathways may also be implicated. High parental care and parenting characterised by a combination of high care and high behavioural control is associated with higher educational attainment, occupational position and income of the offspring in adulthood, independently of family socio-economic circumstances (Blondal & Adalbjarnardottir, 2009; Dornbusch, Ritter, Leiderman, Roberts, & Fraleigh, 1987; Singh-Manoux, Fonagy, & Marmot, 2006). Education and adult socio-economic position are strongly related to elements of positive mental well-being in adulthood (Diener, Ng, Harter, & Arora, 2010).

Only a handful of studies have examined associations between these aspects of parenting and multiple dimensions of positive mental well-being. In a nationally representative study of men and women aged 25–74 in the US, well-being captured by summing Ryff’s psychological well-being scales was positively associated with recalled or perceived parental care summed across mothers and fathers, with effect sizes being larger in men (Rothrauff, Cooney, & An, 2009). In one of the few studies to relate prospectively assessed measures of parental care and control to later well-being indicators, daughters (but not sons) of non-controlling mothers were found to have higher life satisfaction at age 30 (Flouri, 2004). Father–child relationships were not assessed in that study. Data from Britain’s oldest birth cohort study, the Medical Research Council (MRC) National Survey of Health and Development (NSHD), indicated that perceived parental care was positively associated and parental psychological control negatively associated with several aspects of positive mental wellbeing among midlife women, including Ryff’s purpose in life, self-acceptance and positive relations scales (Huppert et al., 2010). Paternal care and psychological control were more consistently associated with well-being than maternal care and control. Men were not included in that study.

It remains unclear whether mother–child or father–child relationships are equally important for mental well-being. Conceptually, the attachment of the child to the primary caregiver (typically the mother) is the key template for future relationships (Bowlby, 1988). Two small, non-random samples of young people in education showed that maternal care was a stronger correlate of happiness when mother– and father–child relationships were considered together (Cheng & Furnham, 2004; Furnham & Cheng, 2000), and that only maternal care and control were related to offspring’s self-esteem, neuroticism and extraversion (Furnham & Cheng, 2000). Based on the literature on psychological disorders, there is a suggestion that the effect of maternal care and control may be somewhat larger, though this is not a universal finding. In the US National Comorbidity Survey, lower levels of maternal and paternal care were related to adult psychopathology but with larger effect sizes for maternal care (Enns et al., 2002). In contrast, in the NSHD, Rodgers (1996a) examined paternal and maternal care and control scales as correlates of psychiatric symptoms at age 43 and found that maternal care did not contribute independently of the other three scales. However, sizeable intercorrelations between these four scales were noted.

The aim of the current study was to describe associations of well-being indicators in adolescence, mid-adulthood and early old age with perceived parental care, behavioural control and psychological control and to assess the contributions of mother–child and father–child relationships to well-being. We hypothesised that well-being would be positively associated with parental care and negatively associated with parental psychological control at each life stage. Based on previous analysis of parental care in this cohort in relation to affective symptoms in men and women at age 43 (Rodgers, 1996a) and mental well-being in women at age 52 (Huppert et al., 2010), we expected to find that paternal care would be more strongly related to well-being than maternal care in mutually adjusted models.

## Methods

### Study population

The NSHD (Kuh et al., 2011) was originally established to investigate the cost of childbirth and the quality of associated care in the immediate post-war years. A sample of 5362, comprising all single births within marriage to families of non-manual and agricultural occupation and a random one in four sample of single births within marriage to families of manual occupation, was recruited from all births registered in 1 week of March 1946 in England, Wales and Scotland. Data have been collected on 23 occasions from birth with the latest data collection in 2006–2011.

### Positive mental well-being

Positive mental well-being in childhood was based on teacher ratings when study members were aged 13 and 15 years, using a forerunner of the Rutter A Scale (Rutter, 1967). Four of these items were positively worded: ‘very popular with other children’ (age 13 years only), ‘unusually happy and contented’, ‘makes friends extremely easily’ and ‘extremely energetic, never tired’. Each of these items was scored as 1 representing the relevant positive rating or 0 representing an average or negative rating. These ratings were summed and grouped into none, one and two or more positive ratings, which yielded one large group with no positive ratings, and two modest and approximately equal-sized groups, respectively (Richards & Huppert, 2011).

Life satisfaction at age 36 was captured with a single item with binary response ‘Would you say that, on the whole, life has been good to you?’ At age 43, concurrently with the Parental Bonding Instrument (PBI), satisfaction with life in the home and family domain was captured with a single item with seven possible response options ‘Looking back, how satisfied are you with what you have accomplished in your home and family life?’ Study members self-completed the 14-item Warwick-Edinburgh Mental Well-being Scale (WEMWBS) at age 60–64. The scale was designed to capture a broad concept of wellbeing, including both eudaimonic and hedonic aspects, and includes items on positive affect, satisfying interpersonal relationships and positive mental functioning. Items are worded positively and respondents are asked to indicate how frequently, on a five-point scale, they have experienced each statement over the last two weeks. Statements include ‘I’ve been feeling good about myself’, ‘I’ve been feeling close to other people’, ‘I’ve been interested in new things’ and ‘I’ve been feeling optimistic about the future’. Validation work indicates good construct validity for a single factor structure as well as good criterion validity and test–retest reliability and supports its use in general population samples (Tennant et al., 2007). Where three or fewer items were missing, the individual’s mean for complete items was imputed. Items loaded on a single component were summed using equal weights to create scores which theoretically ranged from 14 to 70, with 70 indicating highest well-being. The Diener Satisfaction with Life Scale was also completed at age 60–64 (Diener, Emmons, Larsen, & Griffin, 1985). The scale captures cognitive-judgemental aspects of well-being using five items, such as ‘In most ways my life is close to my ideal’, ‘I am satisfied with my life’ and ‘So far I have got the important things I want in life’.

The well-being measures up to age 43 are non-standard and pre-date the development and more widespread use of the validated positive well-being instruments now available. It was not possible, using these data, to examine changes in well-being.

### Parent–child relationships

The quality of relationships with the mother and father in childhood were identified from the PBI which was reported retrospectively on a self-completion questionnaire by the study member during a home visit when they were aged 43. Study members were asked how they remembered the attitudes and behaviours of their mother and father in their first 16 years with responses to 24 items for each parent reported on a four-point Likert scale. Ratings from this instrument are stable over long follow-up periods (Wilhelm, Niven, Parker, & Hadzi-Pavlovic, 2005). Previous factor analysis of the items demonstrated three dimensions of parenting which we refer to here as perceived parental care (12 items e.g. ‘Was affectionate to me’ and ‘Praised me’, internal consistency Cronbach’s *α* = 0.93 for father and 0.91 for mother), perceived parental behavioural control (5 items e.g. ‘Let me go out as often as I wanted’ (reverse coded) and ‘Let me do those things I liked doing’ (reverse coded), *α* = 0.75 for father and 0.77 for mother), and perceived parental psychological control (7 items e.g. ‘Invaded my privacy’ and ‘Tried to make me dependent on her/him’, *α* = 0.83 for father and 0.80 for mother) (Huppert et al., 2010). Although some researchers classify participants into combinations of high/low care and control based on cut-points of these continuous scales, analysis in this cohort and others (Huppert et al., 2010; Rodgers, 1996a; Rothrauff et al., 2009; Schofield et al., 2012) did not indicate that the interaction of care and control provided an improved fit to the data. For this reason, we elected to retain separate scales for perceived care and the two forms of control, rather than dichotomise and combine them.

### Covariates

We selected potential confounding variables which have previously been shown to be related to the quality of the child–parent relationships and to either positive mental well-being or psychological distress (Gale et al., 2013; Kiernan & Huerta, 2008; Rodgers, 1996a, 1996b). Parental separation before the study member was aged 16 was derived from parental marital status collected prospectively throughout the childhood years. Childhood social class was based on father’s occupation at age 4 (or age 11 or 15 if this was missing (*n* = 54)) classified according to the Registrar General’s scheme and included in analysis as a continuous variable from 1 to 6 (with 6 indicating the lowest social class). Maternal mental health was captured by the neuroticism scale of the Maudsley Personality Inventory (Shaw & Hare, 1965) when study members were aged 15. Conceptually, offspring’s personality may mediate the association between parent–child relationships and mental well-being, or it may select children into different levels of parental care and control. Study members’ neuroticism and extraversion were measured by the Maudsley Personality Inventory at age 26.

### Statistical analysis

The six PBI scale scores were standardised. Ordered logistic regression (for the outcomes happy/sociable child at age 13–15 and satisfaction with the home and family at age 43) and logistic regression (for life satisfaction at age 36) were used to estimates associations with perceived parental care and control. WEMWBS and Diener SWLS scores were approximately normally distributed and their associations with perceived parental care and control were estimated using linear regression. We first examined the associations between well-being indicators and care and control for mothers and fathers combined (that is, with each scale score summed across both parents). We then examined care and control from each parent separately (model 1). We examined whether associations between well-being indicators and the PBI scales differed by gender. Where gender interactions were not statistically significant (at the 5% level), we present gender-adjusted analysis. We then included paternal and maternal care and control together (i.e. the six PBI scales were mutually adjusted, model 2). We compared the magnitudes of the estimates for paternal vs. maternal care (and paternal vs. maternal behavioural and psychological control) using Wald tests. Finally, we adjusted for covariates of interest: parental separation before age 16, childhood social class, maternal mental health and study member’s neuroticism and extraversion. Regression models were based on 20 multiply imputed data-sets combined using Rubin’s rules, with additional variables included for the imputation model (household crowding at ages 0, 2, 4, 6, 8, 11; father’s and mother’s educational attainment). Outcomes were included in the imputation model but not used for the estimation part of the model. Analyses were conducted in Stata v12SE.

Of the 5362 original cohort members, 250 had died by age 15, 323 by age 36, 361 by age 43 and 718 by age 60–64. Well-being data were complete for 3699 cohort members at ages 13–15, 3322 at age 36, 3262 at age 43 and 1984 (Diener SWLS) or 1976 (WEMWBS) at age 60–64. Those with missing well-being at age 36 recalled lower paternal care and higher paternal psychological control than those with complete well-being data at age 36. Those with missing well-being at age 60–64 recalled higher paternal psychological control compared with those who had complete well-being data at age 60–64. The magnitude of these differences was small (the largest being 0.15 standard deviations of the parental bonding scale) and no other differences in PBI scales were seen for those with and without each well-being indicator.

## Results

Table 1 summarises the five well-being indicators from ages 13–15 through to age 60–64. Women expressed greater satisfaction with their home and family at age 43 but gender differences in well-being were not apparent at other ages. Women recalled higher levels of paternal care and higher behavioural and psychological control from both parents compared with men. The correlation between perceived maternal and paternal care was moderate (*ρ* = 0.47) and did not differ between male and female study members. Similarly, the coefficient for the correlation between perceived maternal and paternal behavioural control was 0.60 and there were no gender differences. The correlation between perceived maternal and paternal psychological control was slightly larger for men than for women (*ρ* = 0.56 for men and 0.48 for women). Parental separation by age 16 was experienced by 5.6% of study members. In childhood, 45% of study members had a father in a non-manual occupation.

**Table 1.** Well-being indicators across the life course, parental care and control in men and women from the MRC National Survey of Health and Development.

	Men % (*n*)	Women % (*n*)	*P* for gender difference
*Teacher-rated happiness/sociability at age 13*–*15*			
0	56.3 (1083)	59.3 (1053)	0.18
1 positive rating	23.7 (455)	21.6 (384)	
2+ positive ratings	20.0 (385)	19.1 (340)	
*Life been good at age 36*			0.14
No	4.7 (78)	5.9 (97)	
Yes	95.3 (1568)	94.1 (1552)	
*Satisfaction with home and family life at age 43*			0.03
Very dissatisfied	0.9 (15)	0.5 (8)	
Dissatisfied	1.9 (31)	1.1 (17)	
Somewhat dissatisfied	3.3 (54)	2.9 (47)	
Neither satisfied nor dissatisfied	3.3 (53)	2.3 (37)	
Fairly satisfied	18.2 (294)	18.6 (300)	
Satisfied	29.0 (468)	26.9 (434)	
Very satisfied	43.4 (701)	47.8 (771)	
	Mean (SD; *n*)	Mean (SD; *n*)	
*Diener Satisfaction with Life Scale at age 60*–*64*	26.9 (5.8; 930)	26.5 (6.2; 1054)	0.1
*Warwick Edinburgh Mental Wellbeing scale at age 60*–*64*	51.6 (7.9; 930)	51.6 (8.3; 1046)	0.9
*Paternal care (scale 0*–*36)*	21.1 (7.4; 1507)	23.2 (8.1; 1479)	<0.001
*Paternal behavioural control (0*–*15)*	13.7 (4.2; 1516)	12.6 (4.5; 1468)	<0.001
*Paternal psychological control (0*–*21)*	3.3 (2.9;1514)	3.9 (3.3;1493)	<0.001
*Maternal care (0*–*36)*	24.7 (6.2; 1543)	25.0 (7.2; 1522)	0.3
*Maternal behavioural control*	13.9 (3.9; 1553)	13.2 (4.1; 1544)	<0.001
*Maternal psychological control*	4.4 (3.4;1557)	4.8 (3.5;1554)	0.008

Note: SD, standard deviation.

Regression estimates based on multiply imputed data were similar in magnitude and direction to those based on complete cases and the latter will not be described further. Associations between well-being at ages 13–15, 36, 43 and SWLS scores at 60–64 and the parental bonding scales are summarised in Table 2. There was no evidence of effect modification by gender so gender-adjusted analyses are presented. Higher levels of combined parental care were associated with higher well-being at ages 36, 43 and 60–64. Higher levels of combined parental psychological control were associated with poorer well-being at all ages (see also Table 3 for results for the WEMWBS scale). Associations between parental behavioural control and well-being were inconsistent in magnitude and direction across the different well-being indicators. In further analysis, we found no evidence of a non-linear association between well-being and behavioural control (data available on request).

**Table 2.** Associations between the PBI and four well-being indicators.

	Happiness/sociability at age 13–15 (*n* = 3699 imputed)		Life been good at age 36 (*n* = 3295 imputed)		Satisfaction with home and family at age 43 (*n* = 3230 imputed)		Diener Satisfaction With Life Scale at age 60–64 (*n* = 1984 imputed)	
	Values are odds ratios (95% confidence interval)		Values are odds ratios (95% confidence interval)		Values are odds ratios (95% confidence interval)		Values are regression estimates (standard errors)	
	Model 1	Model 2	Model 1	Model 2	Model 1	Model 2	Model 1	Mode 2
*Combined care*	1.02 (0.97,1.07)		1.22 (1.10,1.37)**		1.14 (1.08,1.19)**		0.62 (0.10)**	
*Combined behavioural control*	1.00 (0.95,1.05)		1.01 (0.90,1.13)		0.97 (0.93,1.02)		−0.21 (0.10)*	
*Combined psychological control*	0.95 (0.90,0.99)*		0.83 (0.76,0.92)**		0.95 (0.91,0.99)*		−0.09 (0.09)	
*Paternal care*	1.08 (0.99,1.19)	1.13 (1.02,1.26)*	1.36 (1.12,1.66)*	1.27 (1.00,1.61)*	1.26 (1.16,1.36)**	1.26 (1.15,1.38)**	1.18 (0.17)**	1.16 (0.20)**
*Paternal behavioural control*	1.03 (0.92,1.14)	1.07 (0.94,1.23)	0.97 (0.79,1.19)	0.95 (0.73,1.23)	1.00 (0.92,1.09)	1.11 (1.00,1.24)	−0.39 (0.17)*	−0.28 (0.23)
*Paternal psychological control*	0.95 (0.87,1.04)	0.98 (0.88,1.10)	0.73 (0.62,0.87)**	0.77 (0.63,0.94)*	0.93 (0.87,1.00)	0.97 (0.90,1.06)	−0.10 (0.16)**	0.02 (0.20)
*Maternal care*	0.97 (0.88,1.05)	0.92 (0.83,1.03)	1.26 (1.03,1.53)*	1.16 (0.93,1.44)	1.13 (1.05,1.23)*	1.03 (0.94,1.12)	0.58 (0.17)**	0.07 (0.19)
*Maternal behavioural control*	0.96 (0.87,1.06)	0.93 (0.81,1.05)	1.01 (0.82,1.23)	1.07 (0.83,1.38)	0.90 (0.83,0.97)*	0.85 (0.77,0.95)*	−0.41 (0.18)*	−0.15 (0.22)
*Maternal psychological control*	0.92 (0.84,1.01)	0.93 (0.83,1.04)	0.81 (0.67,0.96)*	0.90 (0.73,1.10)	0.91 (0.85,0.98)*	0.92 (0.85,0.99)*	−0.16 (0.15)	−0.22 (0.19)
Wald test for difference in coefficients for paternal and maternal care		*p* = 0.03		*p* = 0.6		*p* = 0.006		*p* = <0.001
Wald test for difference in coefficients for paternal and maternal behavioural control		*p* = 0.2		*p* = 0.6		*p* = 0.007		*p* = 0.9
Wald test for difference in coefficients for paternal and maternal psychological control		*p* = 0.6		*p* = 0.3		*p* = 0.4		*p* = 0.4

Notes:

^**^ *p* < 0.001

^*^ *p* < 0.05.

Model 1: includes care and control from one parent.

Model 2: includes gender, care and control from both parents (mutually adjusted).

**Table 3.** Associations between the PBI and Warwick Edinburgh Mental Well-being scale at age 60–64.

	*Warwick Edinburgh Mental Well-being at age 60–64* (*n* = 1976 imputed)	
	Values are regression estimates (standard errors)	
	Model 1	Model 2
*Paternal care*	1.15 (0.28)**	1.03 (0.31)**
*Paternal care* *×* *female*	−0.85 (0.39)*	−0.85 (0.39)*
*Paternal behavioural control*	−0.00 (0.23)	−0.13 (0.30)
*Paternal psychological control*	−1.00 (0.20)**	−0.72 (0.25)*
*Maternal care*	0.84 (0.23)**	0.26 (0.26)
*Maternal behavioural control*	0.04 (0.24)	0.25 (0.30)
*Maternal psychological control*	−0.86 (0.21)**	−0.51 (0.25)*
*Combined care*	0.83 (0.13)**	
*Combined behavioural control*	0.07 (0.13)	
*Combined psychological control*	−0.61 (0.12)**	
Wald test for difference in coefficients for paternal and maternal care: men		*p* = 0.001
Women		*p* = 0.1
Wald test for difference in coefficients for paternal and maternal behavioural control		*p* = 0.4
Wald test for difference in coefficients for paternal and maternal psychological control		*p* = 0.8

Notes:

^**^ *p* < 0.001

^*^ *p* < 0.05; SE standard error.

Model 1: includes care and control from one parent.

Model 2: includes gender, care and control from both parents (mutually adjusted).

When the relationship with each parent was considered separately (Table 2, model 1), there was a consistent pattern of higher paternal care and higher maternal care (the latter with the exception of age 13–15) being associated with greater well-being. There was also a general trend of higher paternal and maternal psychological control being associated with poorer well-being when parents were considered separately, although this did not achieve formal statistical significance at all ages. Estimates from model 2 (which included care and control from both parents) were broadly similar, although adjustment for paternal care and control considerably attenuated the associations between maternal care and well-being at ages 36, 43, and 60–64 years, and between maternal psychological control and well-being at age 36. Post-estimation Wald tests revealed that the magnitude of the paternal care estimate was larger than that of the maternal care estimate at ages 13–15, 43 and 60–64 years. The magnitude of the maternal psychological control estimate was statistically significantly greater than that of the paternal psychological control estimate at age 43.

Gender modified the association between paternal care and WEMWBS scores at age 60–64, such that the positive association was larger in men than in women (Table 3, model 1). Paternal psychological control was negatively associated with WEMWBS scores. These associations remained on adjustment for maternal care and control (Table 3, model 2). In the model which considered only the mother–child relationship, maternal care was positively and maternal psychological control negatively associated with WEMWBS score. However, the association between maternal care and WEMWBS score was fully attenuated on adjustment for paternal care and control. Wald tests confirmed a larger magnitude of association between paternal care (compared with maternal care) and well-being in men only (*p* = 0.001).

Positive associations between paternal care and well-being at ages 13–15, 43 and 60–64 years remained on adjustment for childhood social class, parental separation, mother’s mental health, extraversion and neuroticism at age 26, and the other five PBI scales (Table 4). Inverse associations between paternal psychological control and two well-being indicators (at age 36 and WEMWBS score at age 60–64) also remained on adjustment for these covariates. Inverse associations between maternal behavioural control and well-being at age 43 and between maternal psychological control and well-being (at age 43 and WEMWBS score at age 60–64) also remained. More disadvantaged childhood social class was associated with lower likelihood of positive happiness/sociability at age 13–15 and of endorsing that life had been good up to age 36. It was not associated with the well-being indicators at later ages. As expected, greater extraversion at age 26 generally showed positive associations with mental well-being and greater neuroticism was associated with less positive mental well-being. However, personality did not substantially attenuate the associations of interest.

**Table 4.** Fully adjusted associations between the PBI and well-being indicators from age 13–15 to age 60–64.

	Happiness/sociability at age 13–15 (*n* = 3699 imputed)	Life been good at age 36 (*n* = 3295 imputed)	Satisfaction with home and family at age 43 (*n* = 3230 imputed)	Diener Satisfaction With Life Scale at age 60–64 (*n* = 2214 imputed)	Warwick Edinburgh Mental Wellbeing at age 60–64 (*n* = 1976 imputed)
	Odds ratio (95% CI)	Odds ratio (95% CI)	Odds ratio (95% CI)	Regression coefficient (SE)	Regression coefficient (SE)
*Paternal care*	1.11 (1.00,1.23)*	1.20 (0.95,1.52)	1.24 (1.13,1.36)**	1.08 (0.20)**	0.94 (0.30)**
*Paternal care* *×* *female*					−0.67 (0.38)
*Paternal behavioural control*	1.05 (0.92,1.20)	0.94 (0.72,1.21)	1.10 (0.99,1.23)	−0.32 (0.23)	−0.22 (0.30)
*Paternal psychological control*	0.99 (0.88,1.10)	0.78 (0.61,0.95)*	0.98 (0.90,1.07)	0.07 (0.19)	−0.67 (0.24)*
*Maternal care*	0.91 (0.82,1.02)	1.14 (0.92,1.41)	1.02 (0.93,1.12)	0.02 (0.19)	0.15 (0.25)
*Maternal behavioural control*	0.94 (0.82,1.06)	1.03 (0.80,1.33)	0.86 (0.78,0.96)*	−0.13 (0.22)	0.28 (0.30)
*Maternal psychological control*	0.94 (0.84,1.05)	0.93 (0.76,1.14)	0.92 (0.85,1.00)*	−0.20 (0.18)	−0.48 (0.24)*
*Female gender*	0.94 (0.82,1.08)	0.95 (0.67,1.34)	1.26 (1.09,1.45)*	−0.12 (0.28)	0.68 (0.38)
*Childhood social class*^a^	0.94 (0.89,0.99)*	0.81 (0.71,0.91)*	1.04 (0.99,1.10)	0.08 (0.10)	−0.07 (0.14)
*Parental separation*	1.07 (0.81,1.41)	0.83 (0.46,1.48)	1.09 (0.82,1.45)	0.48 (0.58)	0.88 (0.78)
*Mother’s neuroticism*^b^	1.00 (0.96,1.04)	0.90 (0.81,0.99)*	1.00 (0.96,1.05)	−0.06 (0.10)	−0.33 (0.12)*
*Extraversion at age 26*	1.09 (1.07,1.12)**	1.02 (0.96,1.07)	1.05 (1.03,1.08)**	0.12 (0.04)*	0.29 (0.06)**
*Neuroticism at age 26*	1.00 (0.98,1.02)	0.91 (0.86,0.95)**	0.97 (0.95,0.98)**	−0.20 (0.04)**	−0.28 (0.05)**

Notes:

^**^ *p* < 0.001

^*^ *p* < 0.05; CI confidence interval; SE standard error.

^a^ Based on father’s occupation with higher values indicating more disadvantaged class.

^b^ Based on Maudsley Personality Inventory with higher values indicating greater neuroticism.

## Discussion

We have shown that recalled parental care and psychological control are associated with positive mental well-being at several points in the life course. Associations between well-being and parental behavioural control were generally not found, though we note that this construct was captured by only five items and had acceptable though somewhat lower internal consistency than the other two parental bonding scales considered here.

We hypothesised that well-being would be positively associated with parental care and negatively associated with parental psychological control. This hypothesis was supported. Combined levels of parental care from both parents were associated with higher well-being throughout adulthood and combined levels of parental psychological control with lower well-being at all ages. The same patterns were seen for paternal and maternal care and psychological control when each parent–child relationship was considered separately. In models which included the relationship with both parents, associations were attenuated, especially for maternal care and control. In interpreting these mutually adjusted estimates, it is important to note that our models estimated the statistically independent associations of the six parental bonding scales but in reality, these are not independent. There is moderate correlation between mother’s and father’s care and control in this study and others’ (Belsky & Fearon, 2004; Martin, Ryan, & Brooks-Gunn, 2007), and other work indicates concordance in mother’s and father’s parenting is itself associated with children’s cognitive development (Van Bakel & Riksen-Walraven, 2002).

The PBI has sometimes been used to categorise participants into groups described as having an authoritative parenting style (that is, high care and high control), indulgent style (that is, high care and low control) or authoritarian style (that is, low care and high control). Although a combination of high care and high behavioural control is theoretically optimal, several studies find, as we did, that high care is beneficial for well-being, self-esteem and social competence irrespective of the level of control (García & Gracia, 2009; Rothrauff et al., 2009; Schofield et al., 2012). It has been proposed that caring parenting, even in the absence of parental behavioural control, may encourage children to model their parents’ supportive, prosocial behaviour, and that the school and other social environments beyond the family may provide discipline and control (Rothrauff et al., 2009).

We found some evidence that paternal care was more strongly associated with well-being than maternal care. The relative importance of the father–child and mother–child relationships for well-being in adulthood has previously been assessed in only three studies. The largest of these highlighted the importance of paternal care for Ryff’s well-being scales and used data from a subsample of women from the same cohort as the current study (Huppert et al., 2010). Our results showed that associations between paternal care and well-being at ages 13–15, 43 and 60–64 years were larger in magnitude than associations between maternal care and well-being at these ages and are in accordance with Huppert and colleagues. However, at age 43 (but not at other ages), we found that maternal psychological control was more strongly related to well-being than paternal psychological control. Two smaller, non-representative studies found greater importance of maternal compared with paternal care for happiness in young adulthood (Cheng & Furnham, 2004; Furnham & Cheng, 2000). One reason for the differing findings may be that studies used different outcomes. The importance of maternal vs. paternal care may depend on the outcome of interest. Others have noted the equal importance of father’s and mother’s care for infant and childhood outcomes (Boyce et al., 2006; Flouri & Buchanan, 2003), though through different mechanisms (Grossman et al., 2002). Another explanation may be that the perceived relationship with one’s mother is more important at particular life stages, possibly related to one’s own experience as a parent. This is speculative, however, and the current study is limited in not being able to separate the outcome assessment from the measurement age.

For four of the five well-being indicators, we found no evidence of effect modification by gender. For mental well-being measured by the Warwick–Edinburgh Mental Well-being scale at age 60–64, paternal care was positively associated in both genders, but especially in men. Scholars have suggested that identification with the same-gender parent would be greater and that this would encourage sons (daughters) to internalise the behaviours, values and cognitions of their fathers (mothers) (Basuil & Casper, 2012; Hoeve et al., 2009). In this way, boys may be more likely than girls to internalise their fathers caring, supporting and warm attitudes in ways which improve their own interpersonal functioning and affect, as captured by the Warwick–Edinburgh Mental Well-being scale. It has been suggested that child gender may be particularly important to consider when examining father–child relationships because there may be greater flexibility in the paternal role compared with the maternal role, and because fathers (more so than mothers) may respond in different ways to sons and daughters (Parke, 2002).

### Limitations of the current study

Parental care and control were reported retrospectively by the study members themselves when they were aged 43, at which time associations with well-being were strongest and may plausibly be due to memories of parental care and control being influenced by current well-being. Nevertheless, we have considered a range of well-being indicators and not all were completed concurrently with the PBI, suggesting that the associations seen at age 43 are not simply due to reporting or recall bias. Recalled parental bonding on this instrument shows a high level of agreement between twins and other siblings in other studies (Mackinnon, Henderson, & Andrews, 1991; McCrae & Costa, 1988) and correlates in the expected direction with prospectively assessed childhood variables in this cohort (Rodgers, 1996a), thus providing some validation for its retrospective administration. However, relationships with parents and the perceptions of those relationships may change with age and we are unable to account for this. We do not have information about parental care and control in specific phases of the first 16 years of life. In particular, the effect on psychological distress of parental control may be stronger in critical childhood periods (Rutter, 1995) and appears to vary between childhood and adolescence in some studies (Verhoeven, Bogels, & van der Bruggen, 2012). Father’s care has been theorised to be more closely related to positive psychological development in adolescence, whereas mother’s care may be more important in childhood (Bögels & Phares, 2008), though evidence is inconsistent (McLeod et al., 2007). The well-being indicators included in earlier years of this study were not validated and reflect a lesser emphasis on positive mental outcomes prevalent at the time. We were unable to look at changes in well-being and, as noted, the indicator used and the age at which the outcome was assessed were confounded. However, our aim was to explore consistency or otherwise of associations between parenting and a range of well-being indicators. Despite the differences in measurement, our findings are consistent in highlighting the potential importance of perceived paternal care. The extent to which these findings can be generalised is unclear. Unmarried mothers were not part of the original sampling frame for this cohort and lone parents now make up a larger proportion of all parents than they did in earlier decades (Haskey, 1998). However, it is important to note that recalled parental care and control were not patterned by childhood socio-economic circumstances in this cohort, thus eliminating an important confounding structure that may affect later cohorts (Kiernan & Huerta, 2008). As with all longitudinal studies, those lost to follow-up tend to be more socio-economically disadvantaged and in poorer health than those remaining in the study to early older age, yet the cohort continues to be broadly representative on several key demographics, including occupational social class and employment status (Stafford et al., 2013).

## Conclusion

Our study provides evidence from a large, general population study that the quality of the parent–child relationship, along the dimensions of perceived care and psychological control, may have short and long-term consequences for positive mental well-being. These results indicate that relationships with fathers and mothers which are supportive, affectionate and allow the child appropriate autonomy may promote good psychological functioning across life up to and including the seventh decade. Our findings support the view that the foundations for lifelong well-being begin in the early years, or before (HM Government, 2011), and have identified parent–child relationships as a key part of that foundation. This study shows that there are potential long-term gains to taking better emotional care of children, not only by reducing burden of physical (Stewart-Brown, Fletcher, & Wadsworth, 2005) and mental illness (Rodgers, 1996a), but also by improving population mental well-being. These findings reiterate the need to develop parenting programmes and other initiatives which support the development and maintenance of relationships characterised by high levels of care and low levels of psychological control between children and their fathers as well as mothers.
